# Exploring the usability of an internet-based intervention and its providing eHealth platform in an eye-tracking study

**DOI:** 10.1007/s12652-023-04635-4

**Published:** 2023-05-18

**Authors:** Abdul Rahman Idrees, Robin Kraft, Michael Winter, Ann-Marie Küchler, Harald Baumeister, Ronan Reilly, Manfred Reichert, Rüdiger Pryss

**Affiliations:** 1 Institute of Databases and Information Systems, Ulm University, Ulm, Germany; 2 Department of Clinical Psychology and Psychotherapy, Ulm University, Ulm, Germany; 3 Institute of Clinical Epidemiology and Biometry, University of Wuerzburg, Würzburg, Germany; 4 Computer Science and Associate VP for International Affairs, Maynooth University, Maynooth, Ireland

**Keywords:** eHealth, Digital health, eSano, Usability, Persuasive design, Internet-interventions, Eye-tracking, Think-aloud

## Abstract

The proliferation of online eHealth has made it much easier for users to access healthcare services and interventions from the comfort of their own homes. This study looks at how well one such platform—eSano—performs in terms of user experience when delivering mindfulness interventions. In order to assess usability and user experience, several tools such as eye-tracking technology, think-aloud sessions, a system usability scale questionnaire, an application questionnaire, and post-experiment interviews were employed. Participants were evaluated while they accessed the first module of the mindfulness intervention provided by eSano to measure their interaction with the app, and their level of engagement, and to obtain feedback on both the intervention and its overall usability. The results revealed that although users generally rated their experience with the app positively in terms of overall satisfaction, according to data collected through the system usability scale questionnaire, participants rated the first module of the mindfulness intervention as below average. Additionally, eye-tracking data showed that some users skipped long text blocks in favor of answering questions quickly while others spent more than half their time reading them. Henceforth, recommendations were put forward to improve both the usability and persuasiveness of the app—such as incorporating shorter text blocks and more engaging interactive elements—in order to raise adherence rates. Overall findings from this study provide valuable insights into how users interact with the eSano's participant app which can be used as guidelines for the future development of more effective and user-friendly platforms. Moreover, considering these potential improvements will help foster more positive experiences that promote regular engagement with these types of apps; taking into account emotional states and needs that vary across different age groups and abilities.

**Supplementary Information:**

The online version contains supplementary material available at 10.1007/s12652-023-04635-4.

## Introduction

In a recent report, it was estimated that approximately 971 million people worldwide suffer from at least one type of mental illness (James et al. 2018). In the European Union (EU), approximately 38.2% of the population suffers from some form of mental disorder (Wittchen et al. 2011). The cost of treating depression alone was estimated to be around 118 billion euros in 2004 (Sobocki et al. 2006). Mobile and internet-based interventions can play an important role in providing treatment for people struggling with mental illness or behavioral problems. eHealth platforms delivering internet interventions have the potential to be used in the field of translational medicine by providing patients with access to treatment and resources online. If implemented effectively, this may assist in narrowing the treatment gap for those with mental and behavioural disorders. Furthermore, the data and experiences obtained from the usage of these platforms could be used to develop new treatment methods and more readily connect patients with medical experts (Mediouni et al. 2018). However, a common problem with the adoption of internet and mobile-based treatment options is low patient adherence. In general, adherence in the context of behavioral and medical interventions can be broadly defined as the degree to which patient behavior aligns with the directions of their healthcare providers (Vitolins et al. 2000). As suggested by Kelders et al. (2012) and Short et al. (2015), one way to address this challenge is to apply behavior change theories and persuasive design techniques to maintain user motivation and promote greater engagement in treatment. Persuasive design is defined by Fogg (2009) as the use of computer technologies that aim to change an attitude or behavior without coercion or deception. To gain a deeper understanding of how users interact with internet interventions and eHealth platforms, a research study was conducted to address the overall usability of the eSano application. The main focus of the study was on the usability of a mindfulness intervention and how to improve the application’s user experience. eSano is an eHealth platform for internet and mobile-based interventions (IMIs) (Kraft et al. 2021). More details about eSano are included in the following sections. However, this paper does not evaluate the effectiveness of eSano as this is beyond the scope of this study.

The rest of this paper is organized as follows: Sect. 2 discusses related work, Sect. 3 provides background information regarding internet interventions and eSano, as well as persuasive design and behavior change theories, Sect. 4 describes the usability test in detail, Sect. 5 provides the results of the experiment, Sect. 6 presents the discussion, Sect. 7 describes the limitations of the study, and finally, Sect. 8 concludes the paper and discusses future work.

## Related work

This section provides an overview of usability tests and how they are used to evaluate mental and behavioral interventions and support programs. It provides an outline of the most common methods of measurement included in these studies, as well as gaps in the current body of research. Additionally, this section also discusses the current status of eye-tracking in relation to usability tests for eHealth platforms. Stinson et al. (2010) ran a usability study via interviews and observations for an online self-management program for adolescents with juvenile idiopathic arthritis. The results led to several improvements regarding styling, content and functionality. Another study was conducted to evaluate the usability of *My Way-Advanced*—a web-based self-guided psychosocial program for women with metastatic breast cancer (Beatty et al. 2021). Eight participants were included in the evaluation, which used concurrent think-aloud as a means to gather feedback. Results indicated that the participants found the contents to be helpful, but the study was also able to identify several aspects of the program requiring future improvements. Tiburcio et al. (2016) ran a usability test on a web-based intervention that aimed to reduce substance abuse and depressive symptoms. The usability test included N = 9 health professionals and N = 20 drug users. Questionnaires were used to obtain feedback from the participants. As a result of the study, several content alterations were made with regard to structure and styling. Teles et al. (2021) also ran a usability test of an online training and support program for dementia caregivers, named *iSupport*. The test included 10 dementia patients and 5 caregivers. The mouse and screen movements of the participants were recorded, and they were also supplied with pre- and post-experiment questionnaires. Participants found the program trustworthy and were satisfied with its styling and simplicity. Brunette et al. (2016) ran a study to evaluate the usability of a web-based smoking cessation treatment for smokers with schizophrenia. The study included six people and used think-aloud feedback combined with questionnaires. The majority of the participants were satisfied with the website, but several usability problems were also discovered. Cowen et al. (2002) conducted an experimental study to research the use of eye movement in website usability tests. Participants completed two tasks on four different websites. To understand the difference in navigation between different age groups, Romano Bergstrom et al. (2013) provided usability and eye-tracking data from five different website usability studies. The results revealed that there was a difference in performance for older age groups. In one study, this was reflected in lower accuracy rates, and according to Romano Bergstrom et al. (2013) older participants also needed more time to complete the task. MacHado et al. (2018) proposed a concept for adaptive user interfaces that accounts for individual differences in cognitive load and vision loss, allowing developers and designers to gain deeper insight into the usability of their websites and applications among elderly adults. This framework recommends the use of design structures that permit users to personalize different elements such as font size, color contrast, and navigation in order to better accommodate the personal preferences and needs of older users. In addition, by gathering real-time data on the cognitive and sensory abilities of each user, the application UI can be adjusted to enhance general usability and accessibility. In particular, the elimination of small font sizes and complex navigation structures can enhance the user experience for an older cohort of users. Considering the recommendations of MacHado et al. (2018) and Romano Bergstrom et al. (2013), designers can craft applications that are not only visually appealing but also easy to navigate for users across all spectrums of age groups and abilities. Eraslan et al. (2019) presented an eye-tracking study that examined the differences between autistic and non-autistic people when searching for information on a website. The study included 18 autistic participants and 18 neurotypical participants using six web pages. The study showed that there was no significant difference in the number of correct answers given by the two participant groups. However, autistic people were more likely to spend more time on irrelevant elements. Maramba et al. (2019) published a study to explore the different methods used in usability tests for eHealth applications. According to the authors, questionnaires are one of the most common tools used in usability evaluations. Other common tools include think-aloud and post-experience interviews. Eye-tracking, however, is not yet commonly used. Maramba et al. (2019) explained that this could be due to the cost of equipment, e.g., the eye-tracker. One study mentioned by Maramba et al. (2019) used eye-tracking to check the usability of an online diabetes exercise system. Their results were consistent with our findings in that the majority of internet-based studies of intervention usability do not use eye-tracking for their usability tests.

## Background

eSano^1^ is designed as an internet and mobile-based intervention (IMI) platform (Kraft et al. 2021). IMIs are part of the field of eMental Health, i.e., the use of Internet and information technologies to support and treat users (Riper et al. 2010). This includes the use of digital technologies for health promotion, prevention, and early intervention, amongst others (Riper et al. 2010). Digital health interventions are part of a promising field backed by many efficacy studies, as demonstrated by Christensen (2004), Ebert et al. (2017), Moshe et al. (2021), Schröder et al. (2016). As for eSano, several interventions have already been developed and continue to be delivered via the platform. One such example is CBT-based internet intervention PSYCHOnlineTherapie, which is designed to help users who live with depression and anxiety disorders (Baumeister et al. 2021). Another intervention developed and offered via eSano is iCHIMPS. This intervention aims to help adolescent children of parents struggling with mental health disorders (Dülsen et al. 2022). The current number of eSano active users stands at over 975. This number is expected to grow, with more interventions being developed and later offered on the platform.

However, many IMIs suffer from low adherence rates. A systematic review by Kelders et al. (2012) found that only about 50% of participants adhere to the interventions. Another study on adherence included trials of three different internet-based interventions, which resulted in high intervention dropout rates: 48%, 26%, and 49% for the three trials respectively (Wangberg et al. 2008). To mitigate this, Kelders et al. (2012) suggest the use of persuasive technology. Furthermore, Kelders et al. (2012) suggest employing the Persuasive System Design Principles proposed by Oinas-Kukkonen and Harjumaa (2009). Oinas-Kukkonen and Harjumaa (2009) suggested that system features can be divided into four categories: primary task support, dialogue support, system credibility, and social support. For each category, Oinas-Kukkonen and Harjumaa (2009) proposed persuasive principles, such as tunneling, reduction, and personalization. The results of this study could be used to better understand user needs and for the integration of persuasive design technologies in future eSano releases.

## Materials and methods

This section describes the study conditions and materials used throughout the experiment, including hardware equipment, study design, context, procedures, and workflow.

### Study context

**Fig. 1:**
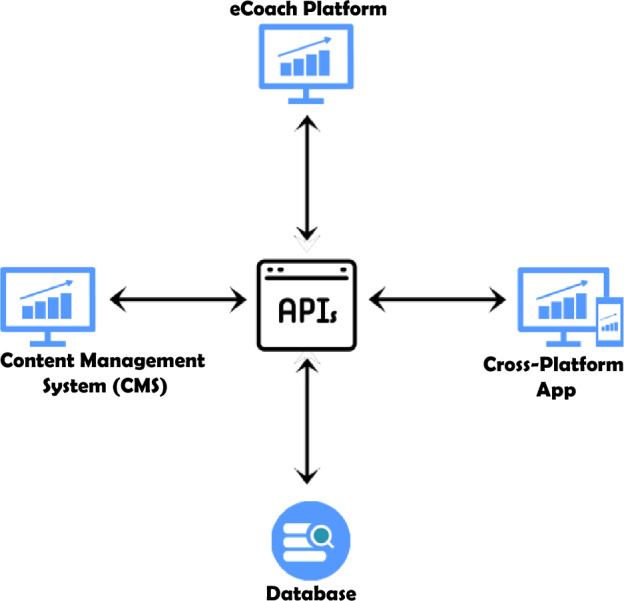
eSano architecture

The participant app of the eSano platform is the tool used to conduct the mindfulness intervention. It was the subject of our study and the focus of the usability test. This experiment was designed to help us locate design flaws in the mindfulness intervention and participant app that could be contributing to a negative user experience. This information could be used to improve the usability of the intervention, further enhance its persuasive features, and increasing user adherence in the future. As shown in Fig. 1, eSano consists of three main parts that are connected via a backend. These three components include a content management system (CMS), an eCoach platform, and a participant app (Kraft et al. 2021). The goal of the CMS is to provide psychologists and researchers with a tool to develop their own interventions. This makes eSano a flexible tool, as new interventions can be developed without making any changes to the backend of the platform. To track the progress of participants during an intervention, the healthcare provider—such as a psychotherapist—can use the eCoach platform to supervise the activity of the user. This way, supervisors can see which modules their users have completed, and provide additional guidance and feedback. The participant app is a cross-platform application that users can access from a desktop or smartphone to complete their modules and interact with their supervisors (Kraft et al. 2021). The backend of eSano helps to serve the underlying functionalities for the three mentioned applications via RESTful APIs. More info can be found at (Idrees et al. 2022).

After logging into the web app, users are presented with the training courses that they subscribe to. To the left, is a side menu containing items such as the user's profile, journals, conversations with the eCoaches and a technical support button. If the user starts a training module, they can find content presented in the form of text, images, video, and audio. Text elements can take different forms depending on the type of content they intent to convey, such as ‘important’, ‘tip’, ‘warning’, or ‘info’. Each text element has its own unique styling. Users also have the possibility to answer questions throughout their training courses. Question types include single choice, multiple choice, sliders, date pickers, and open-ended questions that can be answered via text boxes. Users can stop at any point during their training and continue any time later at the same point that they stopped.

### Study design

All participants were informed in advance about the purpose and procedure of the study. Participation in the study was voluntary, and each participant was informed that he or she could drop out at any time during the experiment. Before participating in the experiment, each participant gave their written consent that the content, procedure, risks, and purpose of the study were adequately explained to them. In addition, participants were informed about data privacy and signed an informed consent form for the use of their data for the purpose of this study. The data collected in this exploratory study will also be used to generate hypotheses for further research, i.e., further research to evaluate and improve the persuasiveness of eSano and the internet interventions offered on the platform.

### Material

**Fig. 2:**
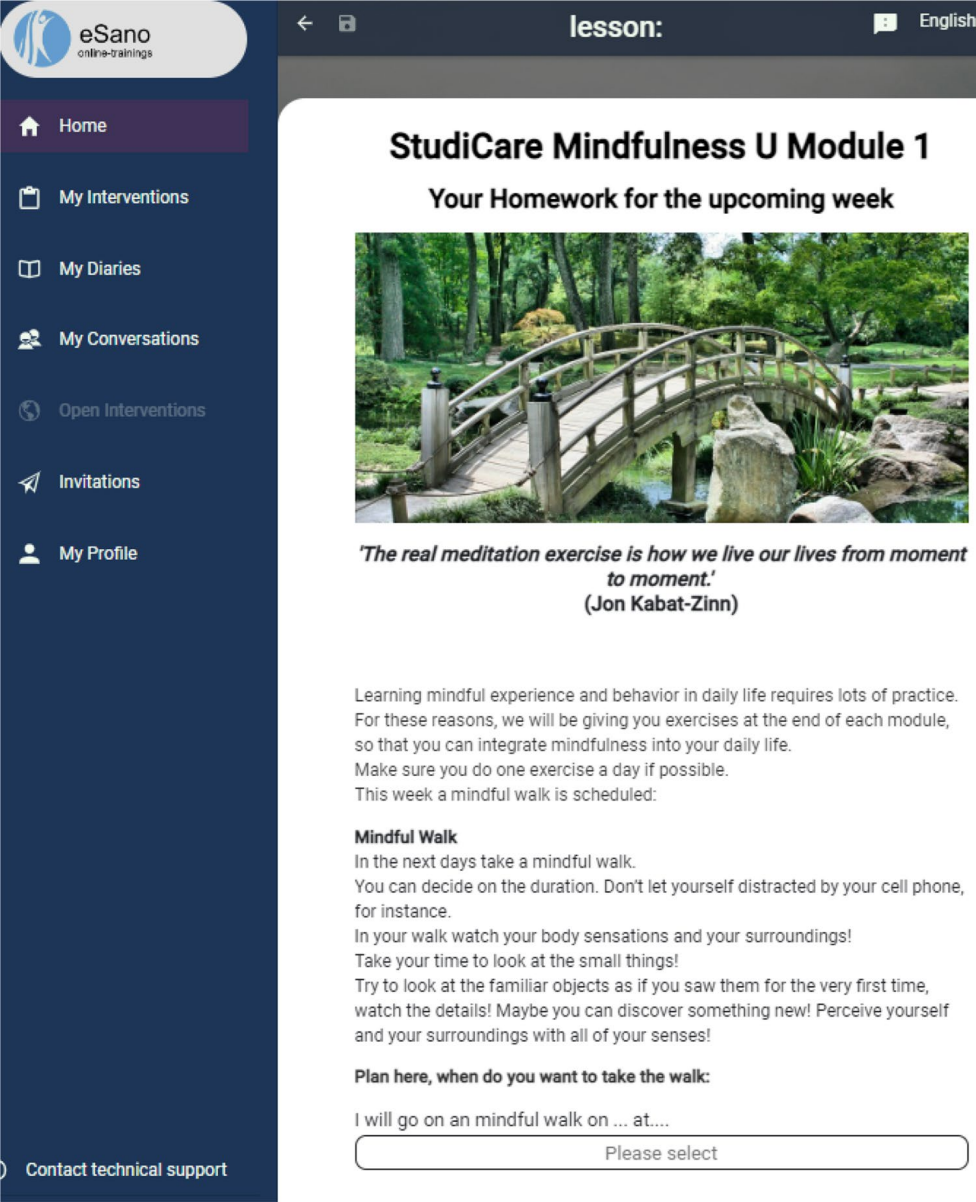
Snippet from one page of the intervention

Participants in the exploratory study were asked to complete the first module of an internet-based preventive mindfulness intervention. It was selected for this study because it is not focused on a specific disorder and contains many elements of eSano found in other interventions, therefore it served as a good example of how eSano typically delivers its interventions to users. This mindful intervention aimed to help students learn how to handle stressful situations and adapt to changing circumstances. The first module was deemed sufficient because the content of the other modules is delivered using the same tools and in the same settings. This module spanned seven pages. It was slightly modified to ensure that it was appropriate for the test. The content of the module was essentially divided into text and images. However, on the last two pages, users were asked several questions to which they were expected to respond using various inputs such as date/time selection, sliders, input fields, and a radio button—depending on the question. Figure 2 shows a snippet from one page of the intervention.

At the start of the module, users received training materials and a brief overview of the content of the entire course. Afterwards, participants who may need psychotherapy were offered further contact options. The module then introduced three fictional students who would accompany participants throughout the intervention. At this point, the module introduced participants to what it means to be mindful and the various benefits of this state. Then, each of the three fictional students described a stressful situation they recently experienced. The module then asked the participants about their day and how mindful they think they were. Once participants reached the next page, they were given their first homework assignment, which was to set a date for a mindful walk in the near future and to download and read materials available as part of the intervention. At the end of the module, participants were given the opportunity to express their opinions and describe how they felt about the module and what they would like to change or improve. Most of the module consisted of text, which meant that users had to read a considerable amount throughout the experiment. The module also contained eight pictures. Three of these pictures showed people, and below each person's picture was a paragraph describing the challenges they faced in maintaining their mental health and well-being. These three images appeared twice in the first module. Another image was also repeated once. In total, the images appeared twelve times throughout the experiment. It is worth noting that two of the eight images also contained multiple sentences/words. The text itself was divided into the following:

- Text on a red background; ‘important’.
- Text on a blue background; ‘info’.
- Text on an orange background; ‘tip’.
- Text under media.
- Text on a white background.

Besides the above-mentioned types, the module also contained text styled in bold and/or italics. Since users of the intervention were faced mostly with the above-mentioned types of text blocks, one sample of each type was selected as an area of interest (AOI). This resulted in five AOIs: AOI 1, AOI 2, AOI 3, AOI 4, and AOI 5. Furthermore, three additional AOIs were selected. These consisted of two images, one of which appears twice in the module. These two images both contained words written inside them. Therefore, AOI 6 and AOI 7 refer to the same image but in two different appearances in the module, while AOI 8 refers to the second image. This divides the AOIs into two categories: text-based (AOI 1, AOI 2, AOI 3, AOI 4, AOI 5) and image-based (AOI 6, AOI 7, and AOI 8).

### Apparatus and experimental settings

The participants’ eye movements were tracked using the open-source 2D eye-tracking platform Pupil Core. It consisted of a wearable eye-tracking headset as well as a software suite. The headset has a sampling frequency of 200 Hz @ 192 × 192px with a latency of 8.5 ms. Furthermore, an outward-facing camera is mounted on the headset to record a video from the perspective of the user with a sampling frequency of 60 Hz @ 1280 × 720px.

A visible microphone was installed on the table next to the monitor to record any thoughts or ideas expressed by the participants while conducting the study. 64-bit Windows 10 was installed on the machine that was used for the experiment. The machine operated with an Intel(R) Core (TM) i5-8350U CPU @ 1.70 GHz and with a maximum frequency of a 1.90 GHz × 64-based processor and 8.00 GB of RAM. A Google Chrome browser was used to provide users access to the participant web app of eSano.

The study was held in a lab at Ulm University, Germany. Participants took part in the study consecutively. Participants were wearing the Pupil Core headset while looking at a 22-inch monitor and sitting in a traditional office environment. The COVID-19 hygiene rules of Ulm University were taken into consideration throughout the study.

### Measures

Eye-tracking techniques can provide information on how users perceive the application during use. However, it has its limitations. (BojkoAgnieszka 2006) indicates that eye-tracking on its own can provide limited results. He argues that it is more beneficial to include other methods alongside eye-tracking while analyzing the user experience. Taking this into account, the following measurement methods were used:

1. *System Usability Scale (SUS) Score*: SUS helps to assess the usability of software and hardware systems (Lewis 2018). Each participant was asked to answer the SUS questionnaire after finishing the assigned task. Scores in SUS go from 1 to 100, with 68 being the average, as indicated by (Sauro 2011).
2. *Application Questionnaire (self-constructed)*: Although SUS could provide a usability score for the participant app, it could not provide any other detailed information. The application questionnaire offered additional insights into the user experience. The questionnaire consisted of 18 questions divided into 4 sections: Usability, User Engagement, Evidence-Based and Visual Design. Participants were presented with a series of positive statements in relation to these topics such as “the app is easy to learn” and “the information is presented in clear and understandable language”. They were then required to select one of the five following responses to each statement: strongly agree, agree, neutral, disagree, and strongly disagree.
3. *Fixation duration*: The average fixation duration can be an indicator of cognitive load (Wass et al. 2013). Longer fixation duration means that the object the user was looking at was either difficult to understand or interesting and engaging (Wang et al. 2014).
4. *Fixation count*: This represents how many fixations the user performed on a particular object (Wang et al. 2014).
5. *Reading pattern analysis*: User reading behavior was analyzed based on the movements of their eyes while reading certain texts. This behavior was compared to other texts in the intervention. This provided more insights into how users perceive different texts based on variables such as text style and text meaning in the intervention (warning, info, tip, etc.). A scanpath analysis was carried out on the text-based AOIs using the technique developed by (von der Malsburg et al. 2015). This is effectively a cluster analysis technique using multidimensional scaling (MDS) that identifies structurally similar scanpaths (Gower 1966).
6. Concurrent think-aloud data in which participants are encouraged to express their thoughts and verbalize their actions while going through the experiment. Participants were informed that they would be recorded so that their verbal feedback could later be analyzed.
7. *Post-experiment interview*: At the end of the experiment, an unstructured interview was conducted. Participants were invited to provide general feedback about their experience using the app and if there was anything they thought could be improved. Further clarification was requested based on the answer given by each participant.

**Fig. 3:**
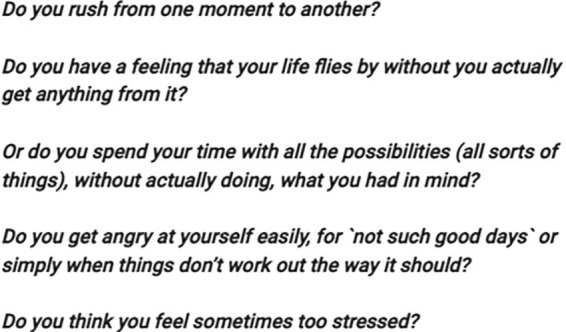
AOI 1—Text in bold and italic

**Fig. 4:**
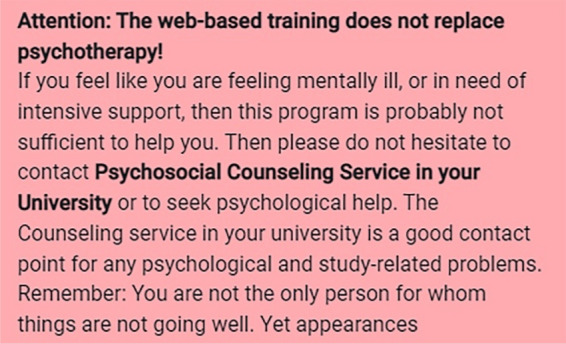
AOI 2—Text on red background

**Fig. 5:**
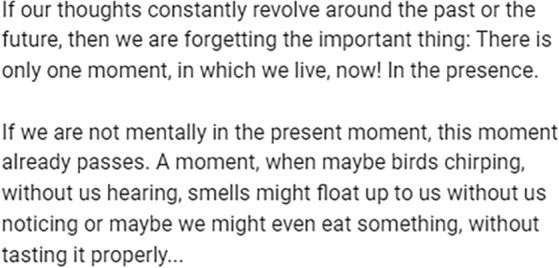
AOI 3—Text on white background

**Fig. 6:**
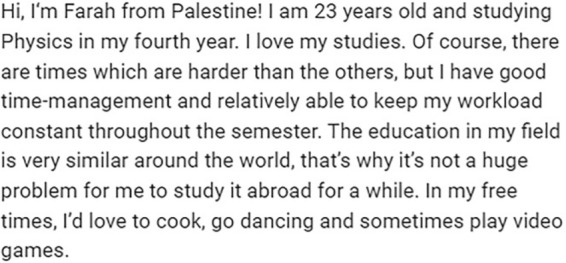
AOI 5—Text under a picture of a person

**Fig. 7:**
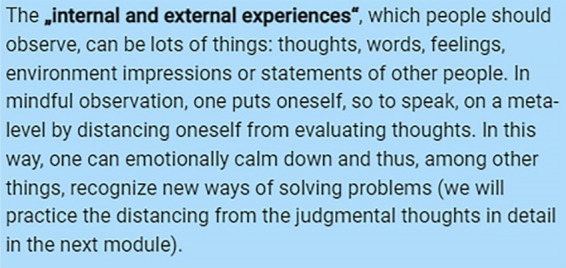
AOI 4—Text on blue background

**Fig. 8:**
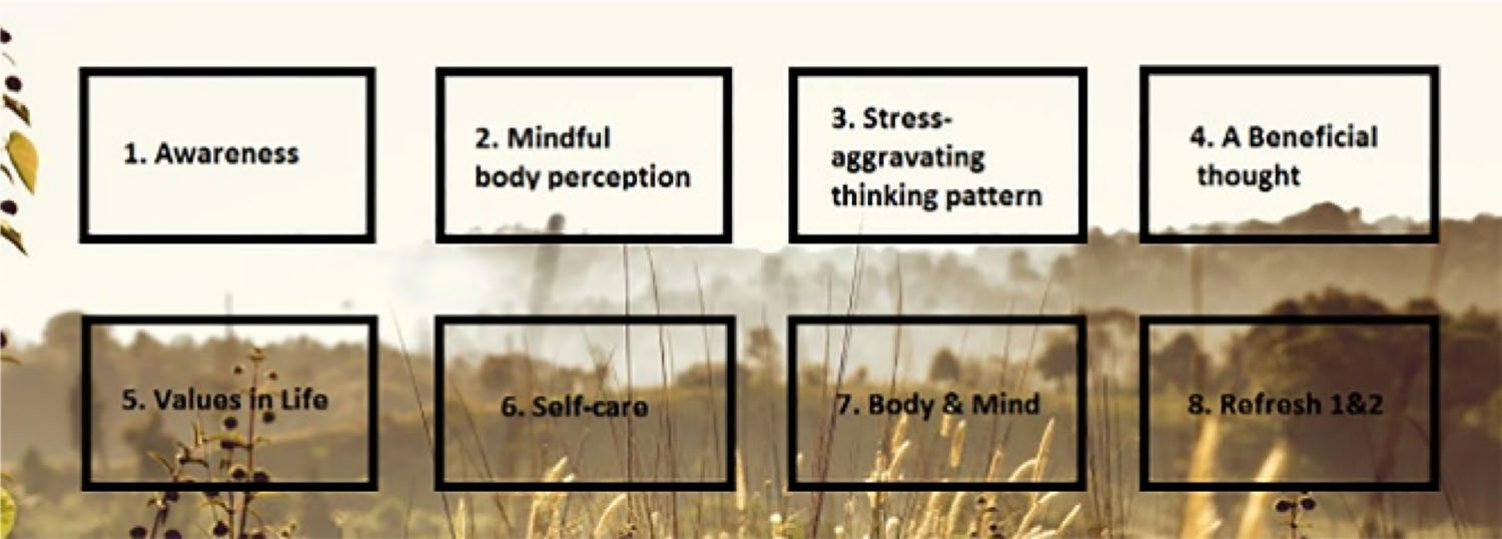
AOI 6—Picture number one

**Fig. 9:**
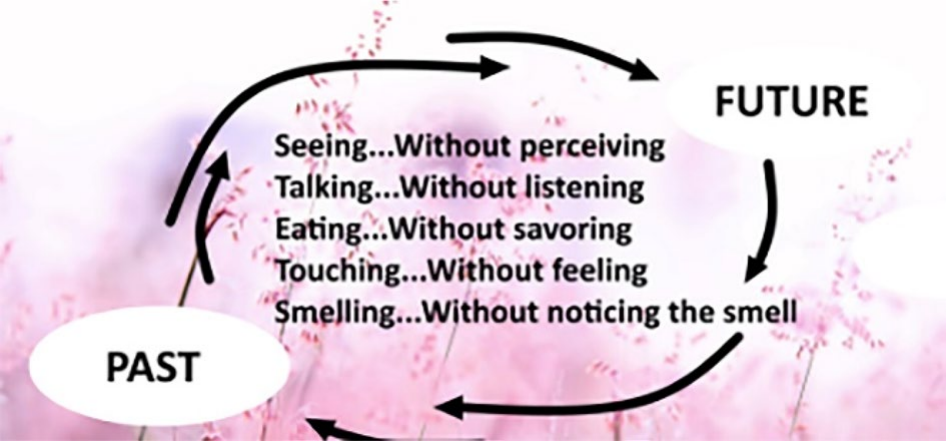
AOI 8—Picture number two

Each participant’s fixation duration and counts were evaluated for the following AOIs in the intervention:

- AOI 1; text block in bold and italic (Fig. 3)
- AOI 2; text on red background (Fig. 4)
- AOI 3; text on white background (Fig. 5)
- AOI 4; text on blue background (Fig. 6)
- AOI 5; text under a picture of a person. The text described an experience as told by the person (Fig. 7)
- AOI 6; picture number one (Fig. 8)
- AOI 7; picture number one (repeated appearance in the intervention, (Fig. 8)
- AOI 8; picture number two (Fig. 9)

### Procedure

**Fig. 10:**
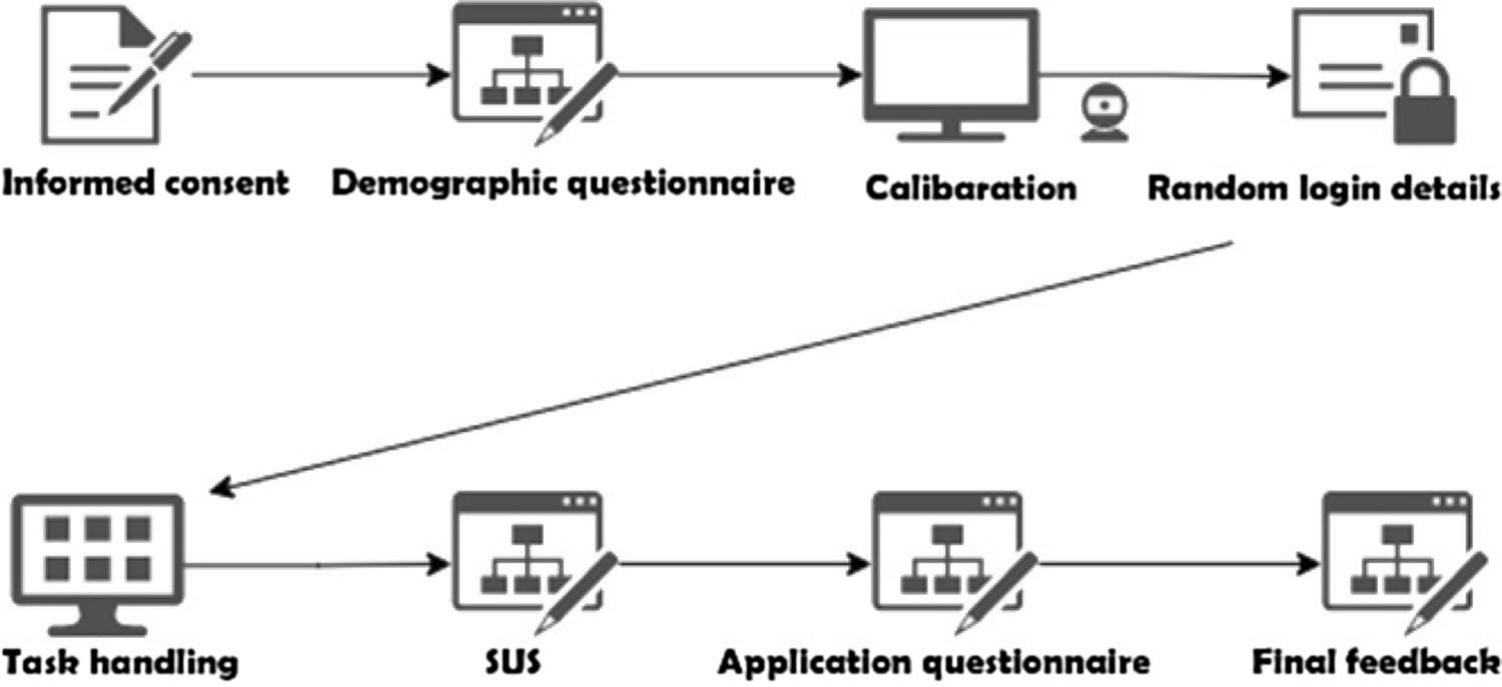
Study workflow

Prior to the exploratory study, a pilot study was run with one participant, to review the study settings and ensure that the study materials and design were valid. To start, each participant was asked to fill in a demographic questionnaire. After that, participants were invited to put on the Pupils Core headset. Once the participant was sitting on the chair comfortably, the headset was calibrated. For this, each participant was asked to use their eyes to follow a black circle on a white background as it moved clockwise around the screen, from one corner into another. This procedure took, on average, about one minute and was repeated for each participant. At this point, participants were given a one-time use random credentials login for the eSano participant app. This was intended to protect the identity of each user and to keep their data anonymous. The study workflow can be seen in Fig. 10. Once participants opened the app, their eye movements and voice were recorded. After users finished the first module, they gave a signal to the experiment supervisor. Eye movement and voice recording were then stopped. In the end, each participant was asked to fill in a SUS questionnaire and an application-specific questionnaire. Afterward, they were asked to participate in an unstructured interview. All the questionnaires were designed using Microsoft Forms and were accessed via a laptop operating Windows 10.

## Results

This section presents the results of the experiment.

### Participants

The study included N = 10 participants. The majority of participants were aged between 20 and 25 (4 participants), or between 26 and 35 (4 participants). The remaining two participants were aged over 35 and 44 years respectively. There were three female participants and seven male participants. Six participants had a master's degree, one had a bachelor's degree, and two had a secondary school diploma. The participants were recruited via student groups at Ulm University. All participants were either undergraduates or post-graduate students, except two participants who were academic workers. All participants had a good command of the English language and were aged 18 years or above. The average processing time for each participant was approximately 60 min.

### System Usability Scale

Participants finished this questionnaire with an average time of 1:01 min. The final SUS score of the mindfulness intervention (first module) – which was accessed by users through the eSano participant's app – was 64.5 with SD = 13.68, Min = 50 and Max = 95. This is lower than the average score for applications in general, which is 68 (Lewis and Sauro 2017). Converting this score into a percentile confers a result of 41 out of 100.

### Application Usability Questionnaire

On average, participants took 03:55 min to complete the questionnaire. Half of the participants agreed or strongly agreed with the statement "the app works well with no problems," while the other half was divided between neutral, disagree and strongly disagree. However, all participants agreed that "the app is easy to learn," indicating that users found the app to be quite usable. Most participants also agreed with the statement that "the information in the app is presented in clear and understandable language," and provided generally positive feedback regarding the visual and aesthetic appeal of the app. However, when asked whether they were able to adjust the color and brightness settings within the app, the responses were divided between 50% neutral, 20% disagree, and 20% strongly disagree, indicating that 90% of participants either could not adjust these settings or were not aware if it was possible to do so. Most participants agreed or strongly agreed with the statement that "the aim and purpose of the app is clear" (90%) and that "the order of the elements makes sense" (80%). However, only half of the participants agreed that "the content is complete but not excessive or irrelevant." Most participants agreed that "the app has interactive tools such as input boxes, checkboxes, etc.," but only 30% of the users agreed with the statement that "the app is fun to use," while 60% were neutral. When asked to give a response to the statement "the app is interesting and encourages repeated use," only 30% of the participants agreed, while 40% were neutral, 20% disagreed, and 10% strongly disagreed. In the user engagement section, most participants disagreed or strongly disagreed that "the app uses elements typical of the game, such as rewards, points, badges, etc." (70%).

### Eye tracking

Table 1 shows the total duration of fixations for each participant on the eight AOIs. Furthermore, Tables 2 and 3 show the average fixation duration and fixation count respectively. It can be observed in all three tables that only two text-based AOIs were never skipped. These were AOI 3 and AOI 5. Conversely, the most skipped text-based AOI was AOI 2. Additionally, AOI 2 received the least number of fixations.

**Table 1:** Total fixation durations in milliseconds

Participants	AOI 1	AOI 2	AOI 3	AOI 4	AOI 5	AOI 6	AOI 7	AOI 8
P1	5106	–	9537	6882	6064	2634	1770	9622
P2	10,150	–	152,033	161,035	85,483	–	–	47,078
P3	–	10,983	17,514	–	25,289	–	–	5907
P4	20,641	32,881	37,126	29,779	19,526	6070	1955	12,385
P5	1676	11,024	20,936	34,481	36,655	14,795	–	31,366
P6	67,758	26,088	69,385	123,754	67,372	142,527	–	46,143
P7	67,396	–	20,980	–	37,177	22,891	–	3041
P8	77,961	80,784	36,016	22,335	42,104	16,134	–	–
P9	119,404	19,636	131,827	160,605	107,051	–	5864	28,930
P10	159,431	93,361	161,724	80,546	135,547	100,116	–	77,378

**Table 2:** Average fixation durations in milliseconds

Participants	AOI 1	AOI 2	AOI 3	AOI 4	AOI 5	AOI 6	AOI 7	AOI 8
P1	160	–	191	215	168	175	161	185
P2	207	–	262	280	234	–	–	254
P3	–	203	177	–	195	–	–	174
P4	193	181	182	197	184	196	196	194
P5	210	235	192	297	211	192	–	192
P6	234	214	212	248	251	311	–	204
P7	215	–	216	–	326	276	–	169
P8	226	232	211	279	271	265	–	–
P9	239	234	264	258	235	–	202	241
P10	254	254	249	278	240	330	–	266

**Table 3:** Fixations count

Participants	AOI 1	AOI 2	AOI 3	AOI 4	AOI 5	AOI 6	AOI 7	AOI 8
P1	33	–	51	33	37	16	12	53
P2	50	–	582	576	367	–	–	186
P3	–	55	100	–	131	–	–	35
P4	108	183	204	152	107	35	11	65
P5	9	48	110	117	175	78	–	164
P6	290	123	329	501	269	460	–	227
P7	314	–	98	–	115	84	–	19
P8	346	349	172	81	156	62	–	–
P9	501	85	500	624	456	–	30	121
P10	628	369	651	291	565	304	–	292

**Fig. 11:**
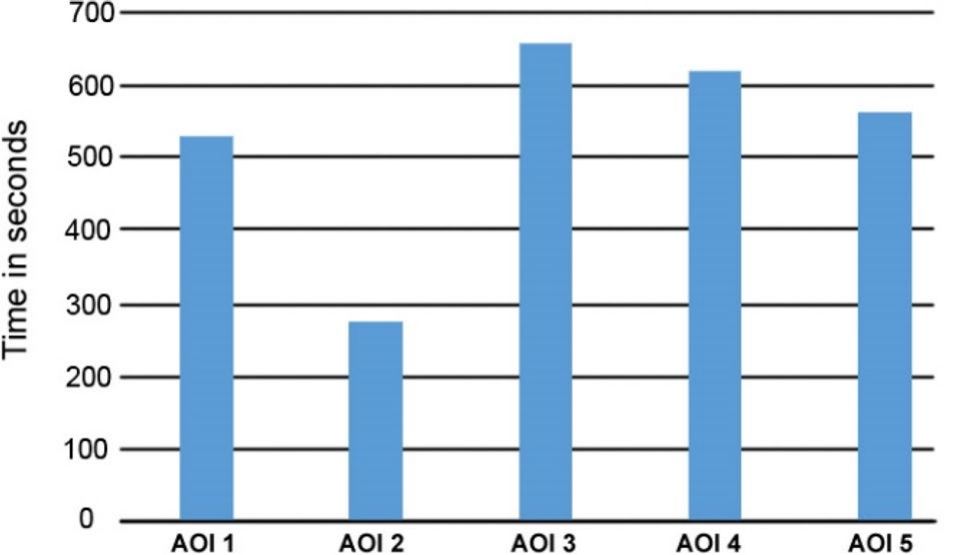
Total fixations spent on each text-based AOI

Figure 11 shows the overall total fixations duration of all the participants on each of the text-based AOIs. It can be observed that users spent the most time on AOI 3, followed by AOI 4 and AOI 5. The speed of reading across all the AOIs seemed to be consistent, with the exception of AOI 2, which was also read the fastest among all the AOIs.

**Fig. 12:**
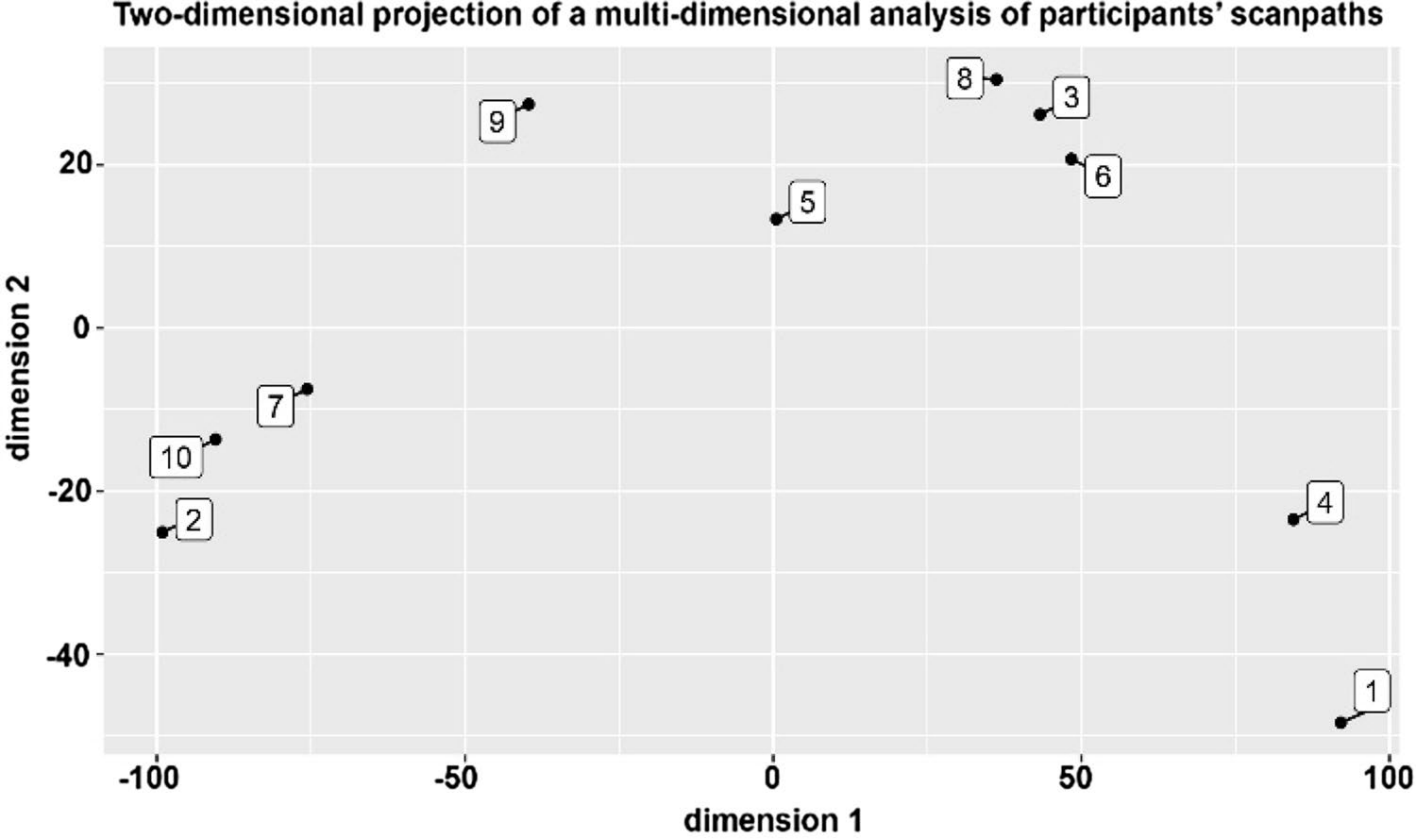
Subject-based two-dimensional projection of a multi-dimensional analysis of participants' scanpaths

**Fig. 13:**
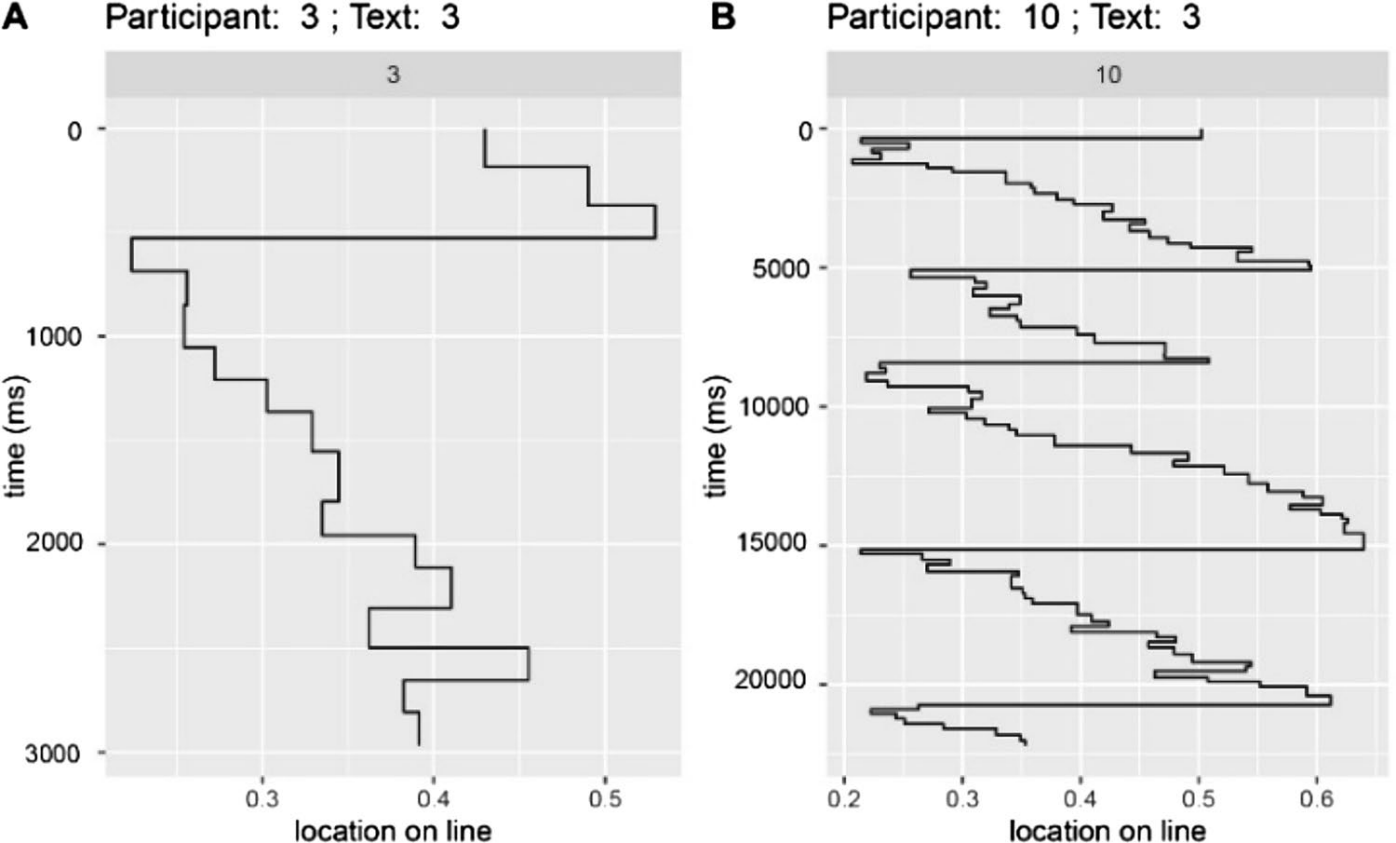
Sample scanpaths for two participants reading the same text AOI. Note that the y-axes are using difference scales

**Fig. 14:**
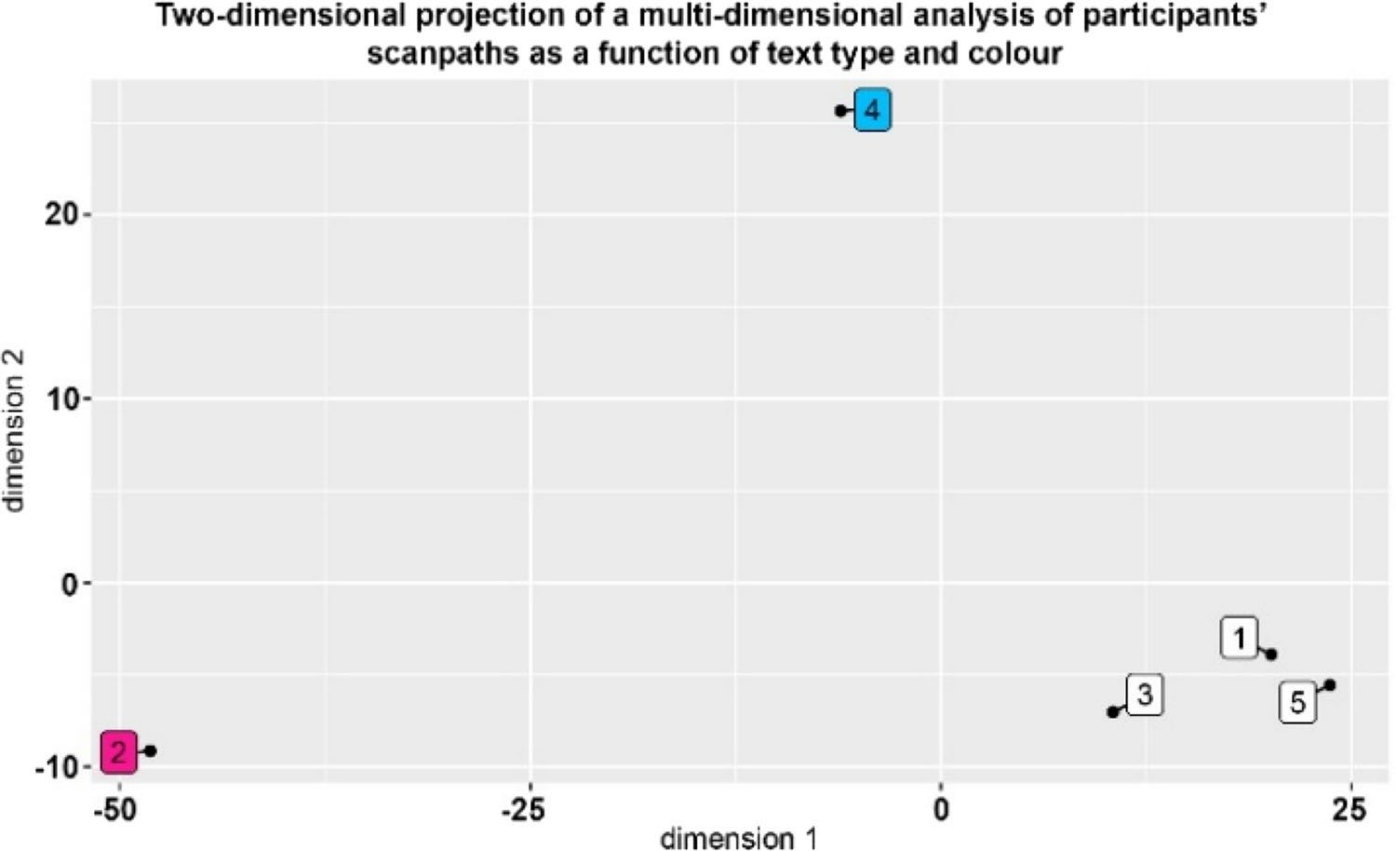
AOI-based two-dimensional projection of a multi-dimensional analysis of participants' scanpaths as a function of text type and background color

In addition to the summary data contained in Tables 1, 2, 3, a pair of sample scanpaths are shown in Fig. 13. The two panels show two participants reading the same text, where the left panel (A) shows the kind of scanpath associated with rapid skimming or truncated reading of the text and (B) shows a pattern associated with more careful reading. Applying MDS to the scanpath data allows us to arrange participants and texts in a two-dimensional space in terms of their mutual similarity (the first components in a principal components analysis (PCA)). In Fig. 12, the relationship among the scanpaths of the 10 participants is represented in a two-dimensional space; the first two dimensions of maximum variation in data. The difference between the two scanpaths shown in Fig. 13 is reflected in the spatial separation of participants 10 and 3. Figure 14 shows a similar projection, but this time for the five texts used. In addition, other observations were made during the experiments:

- 40% of users spent at least half their time answering questions.
- 40% of users partially or fully skipped long texts.
- While answering a question, at least one user was distracted by other questions on the page.
- Push notifications were a source of distraction for some users and they would try to click on them after they appeared.
- 30% of users were confused after they finished their module and were not aware that they had actually finished it.
- Some users missed a few questions at first until prompted by the app, after which they went back to answer them.
- About half the participants did not notice text parts that were hidden behind an expandable
- Menu, resulting in these texts being completely ignored.

Interestingly, scanpath similarity appears to be affected by the background color of the texts, though this may also be due to the information content of the texts in question. Cluster analysis of the text-based AOIs revealed that AOIs with white backgrounds (AOI 1, AOI 3, and AOI 5) had similar scanpaths in comparison to the colored ones (AOI 2 and AOI 4), which can be seen in Fig. 14. The AOI 2 (red background) was the most distinct one.

### Concurrent think-aloud

Not enough think-aloud data was generated by the participants. Several participants started verbalizing their thoughts slowly at the beginning of the experiment and then shortly fell silent afterwards. A gentle reminder was provided but often it led to the disturbance of the participant’s concentration, therefore no more reminders were provided later on. It was possible that some participants were hesitant to verbalize their thoughts because they did not feel comfortable to do so while the study supervisor was in the same lab. Hence, think-aloud procedure did not produce enough data to be analyzed.

### Participant's interview

Participants provided both positive feedback and complaints about various aspects of the application. Some complaints were more about the platform, while others were more intervention-specific. For example, some participants needed some time to understand how to navigate the app. One participant said, "[I]n the beginning, in the first 10 s, I didn't know what my task was". Other participants mentioned that the language settings were not always consistent. For example, warnings were displayed in German even though the app's language settings were set to English. Another common complaint was related to the app's performance. Several users stated that the app was sometimes slow. One participant said, "[T]he loading times are long." Some participants also noted that the app lacked game-like elements that would make it more fun and improve the overall user experience.

Regarding the design of the intervention, some participants expressed concerns about the design and size of the different elements in the app. One participant mentioned, "[M]aybe a thinner font or more space between boxes would be better". Some users complained that the text was too long or that the pictures took up too much space at the expense of the text. For example, "[T]he pictures of the people were so big even though they were not the most important content". It was also noted by several users that in some cases the graphics and text were not always related, even though they were in the same section. In addition to the above feedback, the experiment also revealed some strengths of the platform. One participant mentioned, "[T]he app is easy to use". Another participant said, "[I]t was easy to go through the module.".

## Discussion

Since this was an exploratory pilot study, 10 participants were considered sufficient to assess the usability of the app and to get an overview of the user experience. Even though more participants would have led to more robust results, other studies have shown that similar numbers of participants are sufficient for studies of this type (Beatty et al. 2018) In addition, (Tullis and Stetson 2004) state that the SUS questionnaire can achieve about 75% accuracy with a sample size of 8 and 100% accuracy with a sample size of 12. Kushniruk et al. (1997) also found that when video analysis is incorporated into usability testing of healthcare information systems, a small number of subjects, such as eight, can provide sufficient data. Several participants in the study showed similar reading behavior. If you look at Fig. 12, you can see a clustering of the scanpaths by participant. It can be seen that participants 2, 7, and 10 are close to each other. This is also true for participants 3, 6, and 8, as well as 1 and 4. After analyzing the participants' scanpaths and reviewing the recorded readings of the AOIs, the resulting clustering of the participants' scanpaths can be attributed to the similarity of their reading patterns in terms of temporal and spatial aspects. For example, participants 2, 7, and 10 showed similar reading behaviors, while participant 9 was one of the slower and more engaged participants. Interestingly, it was also observed that text elements within the intervention which had been placed on a red background to highlight their importance were largely ignored by users. An example of this is AOI 2, which informs readers that the current web-based training does not replace psychotherapy and that those who feel they need intensive support should seek psychological help. Nevertheless, this AOI attracted the least attention from participants with the lowest number of fixations. This could indicate that participants were not interested in the information presented in AOI 2. In addition, red is known to be associated with negative emotions such as danger and can increase anxiety for certain individuals (Mehta and Zhu 2009; Ou et al. 2004). Additionally, Tuch et al. (2012) found that users tended to ignore visual elements that were overly complex, including red color elements. To determine whether this behavior is related to the content itself or to the background color, a similar text could be designed with a different background color and participants' eye movement data could be analyzed and compared to the data from this study. These findings could help intervention developers provide more attractive warnings to participants.

The SUS score was 3.5 points below the average. Such a score reflects not only the usability of the first module of the mindfulness intervention but also the way it is delivered through the eSano platform. This means that the score obtained is highly dependent on the content and design of the mindfulness intervention. However, this leaves much room for improvement for both the intervention and eSano itself. To learn more about the experience of the participants using the app, we can refer to the usability questionnaire of the application as well as the unstructured interviews of the participants at the end of their sessions. From the responses of the participants to the application usability questionnaire, one can see that half the participants expressed no issues while using the app. Furthermore, all the participants agreed that the app was easy to learn, indicating a high degree of usability. This was further supported by other statement responses. For example, all the participants agreed that the app was easy to use. This explains why none of the participants asked the study supervisor for help in completing the intervention, except at the end of the module. In addition, all participants were able to complete the task. However, the responses did indicate that users noticed that several of the app settings were not highly customizable. In addition, the majority of users also stated that using the app did not provide any level of entertainment.

Although several complaints were voiced about the design of the intervention, it should be noted that updating the design and layout of the content is relatively easy and does not require updating the base code of the platform. This is due to the flexibility of the platform, which allows intervention designers to make changes to their interventions directly through the CMS. This flexibility allows for easy experimentation and testing with different elements, variations, and styles until user requirements are met. To improve the experience of eSano users, several measures could be considered. First, for the app itself:

1. Improving the performance of the participant's app. (Nah 2004) stated that the average tolerable waiting time for a website to present information is 2 s. Surpassing this threshold could cause users to lose interest in the app, increasing the dropout rate. Also, short load times could allow users with less powerful computers to use the app more easily.
2. Minimizing the number of questions that a user could interact with at any given time. This could help users to stay focused. For example, each question could be contained in a pop-up window, while the rest of the questions are disabled.
3. Provide users with more customization options. As mentioned by Oinas-Kukkonen and Harjumaa (2009), personalization is one of the principles of primary task support in the framework of PSD. Kankanhalli et al. (2021) mentioned several examples of personalization strategies found in health behavior change applications, such as user identification, message personalization, recommendation matching, providing users with descriptive feedback, personalized goal reviewing, personalized rewards, and extra materials, among others. Additionally, consider utilizing Just-in-Time Adaptive Interventions (JITAIs) Nahum-Shani et al. (2018) which can provide the right type of support at the right time while taking into account the changing nature of users’ needs and the overall context.
4. Employ gamification to improve user experience. As indicated by Cechetti et al. (2019), gamification in health-related applications can be effective in increasing users’ engagement without adding complexity.
5. The use of expandable menus is not encouraged since the content below them was ignored by about half the participants.

Regarding the intervention, the following suggestions could improve the experiences of users:

1. Paying more attention to the styling of elements. This includes increasing the readability of the content by improving text styling. For example, ensuring that the font size, style, and color are suitable for the participants’ age range. Furthermore, when choosing the colors of the different elements, one has to carefully consider the influence of these colors on the emotional state of the participants, within the overall context of the application i.e. eHealth. For example, it has been found that red can increase arousal compared to other colors. This could, in turn, affect the performance of users (tasks that are cognitively complex generally require lower levels of arousal.) (Kwallek et al. 1997).
2. Reducing the sizing of some images. Images should not occupy a big portion of the screen without reason. The size of elements should be in harmony.
3. When selecting images, care should be taken to ensure that they represent ideas similar to those discussed.
4. Large text blocks should be broken down into smaller sections.
5. More interactive and fun media elements may replace large text blocks. Stanczyk et al. (2016) compared text against video in computer-tailored interventions for smoking cessation and found that a video-based intervention was more effective in long-term smoke cessation than a text-based intervention.

While using an eHealth platform, users may be experiencing varying emotions. Whether they are in a state of distress or relative calm, it is crucial that the application remains accessible and intuitive, ensuring the effective delivery of treatment. The visual complexity of the user interface can have a critical impact on overall usability, and therefore, designers must consider how UI design may impact users in different emotional states. Taking this into consideration, Stickel et al. (2010) developed the XAOS metric, which sought to measure visual complexity as a factor of usability. The aim of this metric was to assist designers in the optimization of eHealth websites, allowing for the improved delivery of treatment solutions. By considering the findings of this study and implementing further guidance provided by the XAOS metric, and the conceptual framework developed by MacHado et al. (2018), the final design of the eSano participant app could be improved to suit the needs of more users.

## Limitations

Although this study was able to identify several usability weaknesses and user concerns, and also highlighted some of the platform’s strengths, further steps could be taken. Future research will likely be needed to improve the accuracy of the results and gain a more comprehensive understanding of user needs and experiences. Nichols et al. (2019) mentioned that in certain situations, eye-tracking data can be open to more than one interpretation. Therefore, using more evaluation tools alongside eye-tracking may help to provide more insights into user behavior. Furthermore, increasing the number of participants in future studies may yield more robust results and enhance the generalizability of our findings. It should also be noted that the texts for the different AOIs were not the same. Therefore, despite similar word counts, it is not possible to accurately determine how different AOI colors affect the reading behavior of participants. Because the results obtained in this study were intervention dependent, there is still a need to obtain data that is independent of the interventions. One way to accomplish this is to conduct multiple studies, each using a different intervention available on eSano, and then combine the results of these studies to obtain more comprehensive, context-independent findings. It may also prove interesting to spend more time understanding how users perceive different areas on the screen with different background colors, and perhaps even more detailed information about how users interact with AOIs of different background colors. Such insights could help intervention designers focus their users' attention on the most important information in the intervention through better styling. They can also help them understand which elements are typically ignored by users, thus limiting the amount of content ignored by users. Although each participant only engaged in a single session, this work was able to capture multiple requests, suggestions, and feedback from users. However, conducting another study over a longer period of time could provide further insight into how users interact with the application. With this better understanding, designers could improve the persuasiveness of the app and create more lasting user engagement.

## Conclusion and future work

By combining eye-tracking data, the SUS questionnaire, and verbal think-aloud, this study sought to gain a deeper understanding of how users interact with the eSano app while participating in a mindfulness intervention. Upon examining the results of our research, a variety of deductions were made and suggestions were put forward to enhance user engagement with both the application and mindfulness intervention. A key discovery was that prioritizing regular engagement is paramount for higher adherence rates. This could be achieved through careful design and styling of the different elements used in the intervention, considering the varying emotional states and needs of users.

## Supplementary Information

Below is the link to the electronic supplementary material.

Supplementary file1 (XLSX 10 KB)

Supplementary file2 (CSV 1 KB)

Supplementary file3 (CSV 2 KB)

Supplementary file4 (XLSX 9 KB)

Supplementary file5 (PY 1 KB)

Supplementary file6 (PY 1 KB)

Supplementary file7 (TXT 3 KB)

Supplementary file8 (PY 3 KB)

## Data Availability

The datasets generated and/or analyzed during the current study are available from the corresponding author on reasonable request.

## Footnotes

1 https://esano.klips-ulm.de.
